# Measuring early experiences: Challenges and future directions

**DOI:** 10.1016/j.dcn.2025.101637

**Published:** 2025-10-25

**Authors:** Kathryn L. Humphreys, Lucy S. King

**Affiliations:** Vanderbilt University, United States

**Keywords:** Adversity, Stress, Parenting, Caregiving, Early experience, Assessment

## Abstract

The brain’s remarkable plasticity during early development makes it highly responsive to environmental input, with early experiences having lasting effects on functioning and development. Both adversity and variations in normative caregiving experiences influence developmental trajectories. Accurately assessing these diverse experiences is crucial for understanding their role in shaping brain development, yet current measurement approaches face significant challenges that limit our ability to capture the complex, multidimensional nature of children's environmental exposures. This review examines seven key challenges in measuring early experiences: (1) Conflation of exposure and response, (2) Oversimplification of complex experiences, (3) Informant bias and reliability issues, (4) Biomarker overinterpretation and inferential leaps, (5) Limited ecological validity, (6) Genetic confounding, and (7) Limited generalizability across cultures and communities. We discuss how these limitations constrain our understanding of how diverse early experiences shape brain development and propose evidence-based approaches to address each challenge. Emerging frameworks that distinguish between different dimensions of adversity, technological advances in passive monitoring, and genetically-informed research designs offer promising paths forward. By advancing precise, high-dimensional approaches to measuring early experiences, researchers can improve understanding of fundamental neurodevelopmental processes while addressing questions of practical significance in education, mental health, and social policy.

## Highlights

• Seven key challenges limit accurate measurement of early experiences in developmental research.
• Current approaches often conflate environmental exposure with psychological responses to experiences.
• Dimensional frameworks can better capture how different types of experiences affect neurodevelopment.
• Passive monitoring technologies enable ecologically-valid measurement of real-world experiences.

## 1 Introduction

The brain's remarkable plasticity during early development makes it highly responsive to environmental input. Through experience-expectant processes, the fundamental architecture for basic human functions develops, while experience-dependent processes refine and adapt this architecture to each individual's unique environment (Greenough et al., 1987). Because brain development proceeds sequentially, early experiences—whether adverse or enriching—can have lasting effects on functioning. The absence of expected environmental inputs may lead to developmental limitations (Fox et al., 2010), while variations in the quality, timing, and nature of early experiences shape individual differences in cognition, emotion, and behavior (Shonkoff and Phillips, 2000).

Accurately assessing early experiences is crucial for understanding their role in shaping brain development. Both severe adversity (such as institutional rearing, sexual abuse, etc.) and variations within the “normative” range (such as the quality and quantity of parenting behaviors like language input, degree of sensitivity, and enriching activities) influence developmental trajectories (Farber et al., 2022, Hailes et al., 2019, van IJzendoorn et al., 2020). Although research on early adversity and variation in parenting behavior is often treated as separate lines of inquiry, these overlapping constructs are both important for understanding how early experiences affect development.

In this paper, we review approaches to measuring early experiences across developmental research, highlighting opportunities to address current limitations. We discuss frameworks and methodologies that can advance our understanding of how diverse early experiences shape brain development. Finally, we propose guidance for selecting measurement methods appropriate for different research questions and contexts.

## 2 Challenges in current measurement approaches

Current approaches to measuring early experiences face several fundamental challenges that limit our ability to accurately capture the complex, multidimensional nature of children's environmental exposures. These challenges include (1) Conflation of exposure and response, (2) Oversimplification of complex experiences, (3) Informant bias and reliability issues, (4) Biomarker overinterpretation and inferential leaps, (5) Limited ecological validity, (6) Genetic confounding, and (7) Limited generalizability across cultures and communities. In Table 1, we summarize these challenges, why they matter, and approaches to address them. Many research groups are already advancing the field through innovative methods that address these challenges; our aim here is to synthesize the key issues and highlight directions for continued progress. Because no single study can address all of these challenges, we emphasize potential solutions that, taken together across a line of research, can advance understanding of how early experiences shape development.

**Table 1:** Summary of challenges in current measurement approaches, reasons for concern, and strategies to address each challenge.

	**Challenge**	**Why it matters**	**Mitigation strategies**
**1**	Conflation of exposure and response	• Blurs distinction between predictors, mechanisms, and outcomes • Hinders causal inference and model development • Masks interactions between exposure and response	• Clearly separate environmental inputs from biological/psychological responses • Combine objective and subjective measures

**2**	Oversimplification of complex experiences	• Assumes additive effects • Equates different types of experience • Misses timing effects and individual variation	• Use approaches that distinguish core dimensions (threat, deprivation, unpredictability) • Use longitudinal studies with attention to timing and sensitive periods • Measure experiences continuously along adversity–enrichment continuum • Employ cross-species translation to isolate effects of type and timing

**3**	Informant bias and reliability issues	• Limited access to early memories and retrospective bias limit accurate recall • Underreporting of negative behaviors due to social desirability	• Triangulate across multiple informants and methods • Use passive monitoring technologies and EMA • Incorporate administrative data sources

**4**	Biomarker overinterpretation and inferential leaps	• Biomarkers reflect multiple influences beyond target experiences • Blurs distinction between predictors, mechanisms, and outcomes	• Interpret biomarkers as proxies for physiological responses to environment • Anchor biological data in environmental context • Use longitudinal and experimental designs to distinguish environmental “signal” in biomarkers

**5**	Limited ecological validity	• Captures “maximal” rather than “typical” performance • Misses temporal dynamics and real-world variability	• Use passive monitoring technologies and home recording systems to capture experiences as they occur • Combine passive monitoring with EMA to provide context

**6**	Genetic confounding	• Conflates genetic and environmental influences • Gene–environment correlations can be confused for environmental causation	• Use genetically informed designs (twin studies, adoption studies, molecular genetics, within-family designs) • Control for genetic liability using polygenic risk scores

**7**	Limited generalizability across cultures and communities	• Measures developed from culturally homogeneous perspectives may not accurately capture experiences across diverse contexts • May underestimate linguistic enrichment and alternative caregiving practices in non-Western cultures • Fails to capture experiences that disproportionately affect minoritized groups (e.g., discrimination, systemic racism) • Risks perpetuating harm to excluded communities	• Incorporate experiences that disproportionately affect minoritized groups and assess culturally-specific strengths/resilience processes • Develop measures following ethnographic research and in collaboration with community members • Consider how caregiving goals and “optimal” behaviors vary across cultural contexts • Include measures of discrimination and systemic racism as forms of adversity

*Note*. This table summarizes seven key measurement challenges that limit our ability to accurately capture early experiences, their developmental consequences, and evidence-based approaches to address each limitation. EMA = ecological momentary assessment.

### 2.1 Conflation of exposure and response

Clearly defining what constitutes an early experience is a necessary first step in measurement. The term “experience” carries semantic ambiguity that has led researchers to sometimes conflate exposure to an environmental condition with the response to this condition. This conflation is particularly common in research on early adversity and, more broadly, in life stress research (see Harkness and Monroe, 2016).

Both adversity and the related concept of trauma are often confused with the response to those events (i.e., “stress response”). *Trauma*, as defined in the DSM-5, refers to “exposure to actual or threatened death, serious injury, or sexual violence” (American Psychiatric Association, 2013), which represents a narrower set of experiences than adversity broadly. *Stress or stress response* refers to psychobiological responses to challenges in the environment (Szabo and Marian, 2012). When the nervous, endocrine, and immune systems typically involved in the stress response undergo prolonged activation, physiological adaptations known as allostatic load can develop (Danese and McEwen, 2012, Jensen et al., 2017).

Mixing exposure with response creates problems for developing accurate models of how early experiences affect development, as it obscures the distinction between predictors, mechanisms, and outcomes. For example, treating measures of perceived stress as proxies for adversity exposure makes it difficult to determine whether developmental outcomes stem from the objective environment or from individual responses to the environment, which are also shaped by personality, genetics, and mental health (Harkness and Monroe, 2016).

With respect to measures of parenting behavior, this exposure-response issue is less common, but relates to the concept of “child effects” in parenting research, which encompasses how children influence the environment experiences they receive. These effects may play out in transactional models of development in which outcomes result from continuous, dynamic interactions between the child and their experiences (Sameroff and Mackenzie, 2003). For example, children with higher cognitive abilities elicit greater cognitive stimulation from their parents through genetic pathways, but children who receive greater cognitive stimulation also develop better cognitive abilities through environmental pathways (Tucker-Drob and Harden, 2012). If parenting influences development through a transactional model, then it is inaccurate to treat parenting behavior as only a cause and child outcomes as only an effect. Instead, child characteristics and experiences jointly influence developmental trajectories; in fact, some even postulate that genetic variation in children is the primary driver of environmental experiences (Scarr and McCartney, 1983). For example, in autism spectrum disorder, children may show reduced social bids or limited verbal engagement, which can alter the stimulation they receive from caregivers. Parents of children with autism often experience elevated stress (Markfeld et al., 2025), and this stress may affect the amount and quality of language they provide. These processes may reduce children’s exposure to the linguistic input needed for receptive and expressive language development, illustrating how child characteristics and parental responses jointly shape developmental experiences. Brain development, therefore, may both respond to and actively shape early experiences. This suggests that associations between early experiences and neurodevelopment reflect bidirectional processes rather than simple causality.

#### 2.1.1 Potential approaches to address this challenge

Researchers should clearly distinguish between measures of exposure to experiences and measures of psychological or biological responses to those experiences when selecting measures and interpreting findings. This distinction is fundamental to establishing construct validity. Rather than collapsing exposure and response into a single construct, researchers can include both types of measures in analytic models. For example, objective exposure (e.g., documented exposure to violence) and subjective response (e.g., perceived threat) can be modeled as distinct predictors to examine whether responses mediate the effects of exposure or whether responses account for variation in outcomes above and beyond exposure. This approach acknowledges the distinct roles of exposure and response while capturing the ways they jointly shape developmental trajectories. Analytically, this might involve: (1) testing mediation models where subjective responses mediate the relationship between objective exposure and outcomes, (2) examining whether subjective responses explain additional variance beyond objective measures, or (3) testing interactions between objective exposure and subjective responses. The key principle is maintaining the conceptual distinction between what happened (exposure) and how individuals responded to what happened (response) while examining how these processes work together to influence development.

With respect to measures of parenting behavior, researchers should consider how bidirectional effects between parent and child operate not only in developmental processes but also in the act of measurement itself. Standardized observational paradigms such as the Still-Face Paradigm (Tronick et al., 1978) and naturalistic assessments such as LENA recordings (Xu et al., 2009) capture interactions between parents and children, meaning that observed parent behaviors partly reflect responses to child behaviors. Although the quality of such responses is often the focus of research, it is important to recognize that these measures do not solely reflect parent behavior, and to consider whether dyadic, parent-only, or child-only measures best align with the study questions.

### 2.2 Oversimplification of complex experiences

While different early experiences can share common features, they vary in type, timing, duration, and intensity. Because it is challenging to capture this complexity, prevailing measurement approaches tend to oversimplify early experiences. With respect to adversity, the cumulative risk approach has dominated research for several decades. Mirroring the methodology used in the landmark Adverse Childhood Experiences (ACEs) study (Felitti et al., 1998), cumulative risk designs measure adversity by counting the number of discrete forms of adversity an individual has experienced (Evans et al., 2013). At the population level, there is a well-established dose-response association between counts of childhood adversity and later mental and physical health difficulties (Hughes et al., 2017) and several studies have linked cumulative childhood adversity with measures of brain structure and function in childhood and adolescence (Gee, 2021).

While cumulative risk approaches have several benefits (e.g., they are easy to understand and capture multiple experiences), they have important limitations (Machlin et al., 2025). Cumulative risk approaches assume the impact of adversity is additive. In an additive model, the total impact of experiencing emotional neglect and physical abuse is assumed to equal the sum of both individual effects. However, impact may be subadditive, such that risk diminishes with additional adversity beyond a threshold, or nonadditive, such that risk emerges from interactions between adversities. Moreover, cumulative risk approaches treat different types of adversity as equivalent despite the fact that they likely affect distinct neurodevelopmental processes and may lead to different developmental outcomes (McLaughlin et al., 2019).

Research designs that compare groups based on the presence or absence of extreme adversity (i.e., extreme-groups designs) or that examine associations in samples with highly skewed distributions of exposure (i.e., where a small number of participants are highly exposed) also oversimplify complex experiences. While these designs have established that experiences matter—for example, researchers have shown that institutionalized children experience more difficulties across multiple domains than their never-institutionalized counterparts (van IJzendoorn et al., 2020) and that the presence of any type of significant early adversity is associated with elevated risk for mental health difficulties (Vannucci et al., 2025)—they mask important individual variation. Responses to adversity are heterogeneous in nature, with many children experiencing resilience to long-term difficulties while others display severe problems. For example, differences in parenting quality within the context of adversity can shape divergent outcomes among children with similar adversity histories (Humphreys et al., 2022, Labella et al., 2019). Among children exposed to adversity, variation in the nature of exposure explains differential risk (Vannucci et al., 2025). Approaches that reduce experiences to a simple binary, or that fail to sample experiences across the full continuum of enrichment to adversity, obscure this heterogeneity and limit our ability to identify the processes that drive differences in developmental outcomes.

#### 2.2.1 Potential approaches to address this challenge

Several statistical approaches can address the limitations of cumulative risk by modeling nonadditive and interactive relationships, although these approaches require larger sample sizes and may be sensitive to analytic choices. For example, generalized additive models, spline regression, and polynomial terms allow researchers to test for nonlinear effects such as thresholds, inflection points, or diminishing returns (Grimm et al., 2011), while person-centered (e.g., latent class/profile models) (Lanza and Cooper, 2016) or machine learning (e.g., random forests) approaches (Rosenbusch et al., 2021) can identify synergistic or interactive effects among experiences.

Research approaches that distinguish core dimensions of experience thought to influence neurodevelopment through different pathways can address oversimplification (McLaughlin et al., 2021). For example, distinguishing between adverse experiences characterized by threat (presence of negative input such as abuse) and those characterized by deprivation (absence of positive input, such as neglect) enables more precise hypotheses about brain-experience relationships (McLaughlin and Sheridan, 2016). Additional dimensions such as unpredictability (Usacheva et al., 2022), emotional input, and cognitive input (King et al., 2019) may further elaborate these models. Continuous measures of experience allow researchers to characterize how experiences relate to outcomes, including the thresholds at which they exert an impact. Because experiences span a continuum from enrichment to adversity, it is important to capture the presence of positive experiences rather than merely the absence of negative ones. For example, within the domain of deprivation, the absence of overt neglect is not equivalent to the presence of a highly stimulating and nurturing environment.

Longitudinal developmental studies that consider timing and sensitive periods are crucial for clarifying how the effects of experiences unfold across development and whether effects vary based on when experiences occur (Gabard-Durnam and McLaughlin, 2020). Such studies should also capture variation in exposure intensity, duration, and chronicity, as these parameters may moderate developmental impacts. For example, brief early adversity may have different effects than chronic exposure, and the same objective dose of adversity may have different impacts depending on the developmental period during which it occurs. Cross-species translation using parallel measurement approaches can enhance mechanistic understanding by connecting manipulations of the type and timing of experience in animal models to naturalistic variations in human development (Gee, 2021).

### 2.3 Informant bias and reliability issues

Researchers often measure early experiences through retrospective self- and informant-report of exposure to life events, such as self- or parent-reports of lifetime adversity or parent-reports of their own parenting behavior. While these measures offer an efficient way to capture aspects of children's experiences, they are subject to methodological issues that complicate the interpretation of associations with developmental outcomes. Specifically, retrospective measures are limited by individuals’ restricted access to early memories and potential biases in recall (Reuben et al., 2016). Compared to measures that are collected closer to the time of exposure—often labeled “prospective” despite not capturing experiences exactly as they occur—retrospective reports may more strongly reflect subjective appraisal of experiences (Baldwin et al., 2024). “Prospective” and retrospective measures of adverse childhood experiences have the potential to diverge (Baldwin et al., 2021), and relate differently to outcomes of interest such that retrospective reports are more strongly associated with life outcomes that are subjectively assessed, whereas “prospective” measures are more strongly associated with outcomes that are objectively assessed (Reuben et al., 2016).

Because infants and young children are unable to provide verbal reports on their own experiences, researchers frequently rely on parent reports during early development. Parent-reported parenting behaviors remain useful even as children age, as children may lack the insight to evaluate certain dynamics (e.g., parental monitoring or discipline). However, parent reports also introduce challenges. Parents may be unaware of aspects of their children's experiences—particularly those occurring outside the home—and may have difficulty accurately estimating the frequency or quality of their interactions (Morsbach and Prinz, 2006). For example, estimating how often or for how long one provides physical comfort to their child is unlikely to be reported accurately. Experiences defined by the omission of input, such as emotional neglect or other forms of deprivation, may be especially prone to underreporting because they are less salient or consciously recognized. Social desirability bias may also lead to the underreporting of negative parenting behaviors or family dynamics (Bornstein et al., 2015).

#### 2.3.1 Potential approaches to address this challenge

Triangulating measures across multiple informants and methods can increase accuracy and reduce bias, with different techniques addressing different shortfalls. However, the alternatives to retrospective self-report—such as passive monitoring technologies, prospective data collection, and multi-informant approaches—require substantially more resources in terms of staff, long-term funding, and years of data collection, which may limit their feasibility for many studies. Nevertheless, these approaches can provide important advantages when resources permit. Passive monitoring technologies (e.g., LENA wearable devices for language input (Xu et al., 2009), accelerometers for physical activity (Cain et al., 2013), and proximity sensors for social interactions (Salo et al., 2022)) can provide objective measures that reduce reliance on retrospective reports and capture experiences as they naturally occur. Ecological momentary assessment (EMA) using brief, frequent self-reported assessments in natural environments can capture experiences in real-time, reducing recall bias. Administrative data (e.g., electronic health records, social service records) can provide information about certain types of experiences without relying on self-report. These approaches also enhance ecological validity by capturing experiences as they naturally occur (see Challenge 5). Community-level indices (e.g., the Child Opportunity Index; Ferrara et al., 2024) provide another complementary approach. These measures capture structural features of environments that shape children’s experiences and can serve as more objective measures of contextual adversity. However, they lack individual specificity and may underestimate heterogeneity within populations, particularly in communities with high poverty levels. When using parent reports, researchers should acknowledge their limitations and consider incorporating observational methods to provide a more comprehensive assessment.

### 2.4 Biomarker overinterpretation and inferential leaps

In an attempt to obtain objective assessments of early experiences, researchers have increasingly turned to measuring biomarkers (Qian et al., 2024). However, this approach introduces its own set of challenges and risks of inferential overreach. Biomarkers, such as cortisol and inflammatory markers, are influenced by numerous factors beyond the experiences researchers aim to measure. Basal cortisol levels, for example, are shaped by circadian rhythms, physical activity, nutrition, and genetic factors (Stalder and Kirschbaum, 2012), and inflammatory markers are affected by health behaviors (Loprinzi, 2016). Because biomarkers are multi-determined, “signal” related to specific experiences is diluted by other influences. This complexity is evident in meta-analyses showing small or null overall associations between childhood adversity and salivary diurnal cortisol (Perrone et al., 2024) or circulating inflammatory markers (Kuhlman et al., 2020), whereas more robust associations have been identified for salivary cortisol responses to laboratory-based social stressors (Bunea et al., 2017).

In the context of being multi-determined, biomarkers are sometimes inaccurately conflated with the broad construct of “stress.” For example, despite longstanding recognition that measurements of cortisol cannot, on their own, reveal whether a person is stressed (Levine et al., 1989), research groups have recently examined the use of cortisol to monitor psychological stress in psychiatric disorders (Ahmed et al., 2023), to identify stress in daily life (Choi et al., 2014, Ok et al., 2024), and to measure the experience of chronic stress in early childhood (Bates et al., 2017). Cortisol, however, serves essential regulatory functions under normal physiological conditions (McEwen, 2019). Accordingly, its interpretation—and determining whether it reflects stress—requires consideration of temporal patterns and environmental context. Given the multiple influences on biomarker levels, the validity of using them to index stress responses outside of controlled experimental settings is questionable.

Interpreting biomarkers as reflecting stress or the negative consequence of adversity can lead to misleading conclusions about the nature and impact of experiences. Specifically, this risks a problematic reverse inference, where differences in biomarkers observed with adversity are assumed to signal pathology, while those linked with enrichment are assumed to signal optimal development. Elevated cortisol in children from adverse environments may, in fact, reflect positive adaptations to challenging circumstances rather than pathological stress responses (Ellis et al., 2022). Differences in the structure and function of brain regions among children from lower socioeconomic backgrounds may reflect alternative, but not inherently negative, experiences and developmental processes (Ellwood-Lowe et al., 2016). Such differences should not be assumed to represent deficits without evidence of how they relate to function.

#### 2.4.1 Potential approaches to address this challenge

Researchers should interpret biomarkers as proxies for physiological responses to early life experiences rather than direct measures of stress or experiences. When using biomarkers, it is crucial to anchor biological findings in environmental context by simultaneously measuring actual environmental conditions and by considering multiple factors that influence biomarker levels at the time of measurement—including diurnal rhythms, recent stressors that are not part of the target construct, and health status (Stalder et al., 2016). Incorporating repeated measures of biomarkers in tandem with repeated measurement of experiences may clarify whether they are meaningful measures of physiological responses to these experiences and reduce confounding due to stable individual differences (Hruschka et al., 2005). Examining changes in biomarkers such as cortisol in response to experimental manipulations (e.g., the Trier Social Stress Test; Kirschbaum et al., 1993) can also disambiguate signal related to stress responses versus other factors. Overall, these approaches help to avoid overgeneralization, provide more accurate interpretations of biomarker findings, and may yield complementary information about both exposure and response processes.

### 2.5 Limited ecological validity

Objective measures of parenting behavior typically rely on brief, structured, laboratory-based observations. While these methods provide valuable, standardized assessments of parent–child interactions, they are limited in ecological validity. They may capture “maximal performance” rather than “typical performance” of parenting (Humphreys and Garon-Bissonnette, 2025), potentially overestimating the quality of interactions or missing meaningful features of how interactions ebb and flow in everyday life. For example, in structured play settings, mothers consistently provided their infants with language input that was high in both quantity and diversity. In contrast, language input during naturalistic routines was more variable—characterized by dense periods of interaction interspersed with silence (Tamis-LeMonda et al., 2017). Thus, structured observations may miss the temporal dynamics of parenting behavior, producing “both false alarms and misses” in how input is characterized (Tamis-LeMonda et al., 2017, p. 11). The effect of the laboratory environment on measured performance may be confounded by cultural and socioeconomic differences in perspectives on what constitutes desirable behavior (see Challenge 7).

These temporal dynamics may themselves be a meaningful dimension of early experience. The sensitivity of parental input—how attuned it is to the child's moment-to-moment cues and needs—is a critical feature that may moderate the impact of parenting behaviors on developmental outcomes (King et al., 2019). Taking language input as an example, although a greater quantity is often assumed to be beneficial, the match between input and the child's current state (e.g., energy level, bids for interaction) may be equally important. As another example, disciplinary parenting behaviors may have different effects on children's development when engaged in predictably but infrequently (potentially reflecting well-timed limit setting) than when used pervasively (reflecting a stable pattern of harshness or overcontrol).

#### 2.5.1 Potential approaches to address this challenge

Recent advancements in passive monitoring technologies (e.g., wearable devices, home recording systems) are expanding our ability to obtain ecologically valid measures of the early environment and to capture temporal dynamics in children's everyday experiences as they naturally occur. While traditional naturalistic observations involving human observers can provide rich contextual data about real-world behaviors, they are resource-intensive and may be influenced by observer effects. Passive monitoring technologies address some of these limitations by capturing genuine variability in parent–child interactions distributed across different contexts and time scales, enabling researchers to measure early experiences at a scale large enough to robustly examine associations with development. Some of these approaches also address informant bias issues discussed in Challenge 3, as they reduce reliance on retrospective self-report. However, passive monitoring methods have limitations—for example, LENA recordings cannot always clearly determine speaker identity, and proximity sensors cannot distinguish between types of physical contact or their emotional tone. Combining passive monitoring with ecological momentary assessment can provide insight into subjective experiences and contextual factors that passive sensors alone cannot detect. For example, our lab has linked repeated EMA measures of infant placement in daily life (e.g., held, bouncy seat) with continuous language environment recordings to examine how opportunities for receiving adult speech in infancy vary based on physical context (Malachowski et al., 2023).

### 2.6 Genetic confounding

An important methodological challenge that cuts across multiple measurement approaches is the issue of genetic confounding in studies of early experiences and developmental outcomes. Traditional observational studies of parenting and child outcomes may conflate genetic and environmental influences because parents provide both genes and environment to their children. As Harden (2021) noted, many areas of psychology continue to practice ‘epistemological tacit collusion,’ where genetic confounding potentially poses significant problems for inference, but investigators rarely address it in their own work or raise it when evaluating others’ research. This represents an enormous waste of scientific resources, particularly given that genetic differences potentially confound nearly all correlational studies of relationships between individual psychological outcomes and environmental contexts. Children who experience more positive parenting may show better outcomes partly due to shared genetic factors that influence both parental behavior and child characteristics, rather than purely environmental effects (Sherlock and Zietsch, 2018). This issue is particularly problematic because it can lead to overestimation of environmental effects and misguided intervention efforts targeting factors that may not be truly causal.

The challenge of genetic confounding affects virtually all measurement approaches discussed above. Parent reports, observational measures, and biomarkers may reflect both genetic and environmental influences, making it difficult to isolate the causal effects of environmental experiences. Some apparent “environmental” effects may actually reflect children's genetic predispositions that evoke certain responses from their environment through gene-environment correlations (Scarr and McCartney, 1983). For example, children with genetic predispositions toward more challenging temperaments may elicit harsher parenting (Hajal et al., 2015), which may then appear to “cause” behavioral problems in traditional analyses that do not account for genetic factors.

#### 2.6.1 Potential approaches to address this challenge

Genetically-informed research designs can help disentangle genetic and environmental influences and clarify the causal role of environmental experiences. Twin studies, adoption studies, and molecular genetic approaches each offer unique advantages for addressing genetic confounding. For example, adoption studies can compare adopted children to their biological and adoptive parents to isolate environmental effects and examine gene-by-environment interactions (Rutter and Silberg, 2002). Within-family designs can compare siblings who experience different environmental conditions while sharing similar genetic backgrounds (D’Onofrio et al., 2013). When genetically-informed designs are not available, researchers can use polygenic risk scores as covariates to partially control for genetic liability, though this approach captures only a subset of genetic influences. For example, Cuevas et al., (2021) demonstrated that associations between discrimination and anxiety remained significant even after controlling for polygenic scores for anxiety, depression, and neuroticism, suggesting genuine environmental effects. Researchers should acknowledge when genetic confounding cannot be ruled out and interpret associations as correlational rather than causal. Collaboration with existing genetically-informed studies through data sharing initiatives or contributing data to consortia that include genetic information can help address these limitations across a research program.

### 2.7 Limited generalizability across cultures and communities

Many commonly used measures of early experience were developed from a culturally homogeneous perspective—specifically, Western, white, and socioeconomically advantaged—raising concerns about their generalizability across diverse contexts.

Attachment theory has strongly influenced measures of parenting, typically defining optimal care as highly responsive, child-centered, and characterized by mutual exchange, and focused on one or two primary relationships, usually the mother. But these premises are rooted in Western, middle-class culture; in other cultural contexts (e.g., where caregiving is more distributed), care may be expressed differently and behaviors carry different meanings and impacts (Keller, 2018). For example, while research on the language environment highlights the importance of child-directed speech, rates of adult-to-child speech vary widely across communities, and children in some cultures with little child-directed speech do not show language delays (Casillas et al., 2021). Within U.S. communities, measures that exclude multiple caregivers and bystander talk can underestimate linguistic enrichment in low-income families (Sperry et al., 2019).

Commonly used measures of adversity may fail to capture experiences that disproportionately affect minoritized groups (e.g., racial and ethnic discrimination, systemic racism, immigration-related adversity) because they were not grounded in the perspectives of those with lived experience or developed with the participation of affected communities. Although race and ethnicity are often treated as risk factors for disparities in developmental outcomes, less attention is given to experiences associated with race and ethnicity that may underlie these associations (Shonkoff et al., 2021), constraining theoretical models of how developmental pathways unfold. Moreover, specific experiences (e.g., how caregivers communicate with children about race and ethnicity and help them cope with discrimination) may buffer the impact of racism on development (Neblett, 2023). Failing to accurately capture and interpret experiences of parenting and adversity across diverse communities—particularly those disproportionately affected by adversity and also subject to heightened surveillance—limits understanding of the developmental impacts of these experiences and risks perpetuating harm.

#### 2.7.1 Potential approach to address this challenge

Addressing limitations in cultural generalizability requires rethinking both what is measured and how measures are developed. This includes incorporating experiences that disproportionately affect minoritized groups and assessing specific strengths and resilience processes that may buffer the impact of adversity related to discrimination and systemic racism. Researchers should also prioritize culturally informed approaches to measurement, including developing measures following ethnographic research that examines meaning systems and behavioral standards (e.g., caregiving goals and what defines a “good” parent in that culture; Keller et al., 2018) and in collaboration with community members who are the experts in their own environments.

## 3 Guidance for selecting measurement approaches

Given the complexity of early experiences and the variety of measurement approaches available, it is unlikely that any single research study can comprehensively address questions about how the early environment shapes development. Despite decades of research, individual studies typically explain only 5–10 % of variance in outcomes, suggesting that current measures capture only a fraction of the total environmental signal. Large-scale studies, smaller studies that use deep measurement across time and context, and meta-scientific approaches that aggregate data across samples can provide complementary insights. However, the potential of any of these approaches remains constrained by the quality of the chosen measurement approaches and the accuracy of their interpretation. The challenges reviewed above include both issues of identifying the right information to measure (e.g., conflation of exposure and response) and issues of how that information is measured (e.g., informant bias and reliability). Selecting a measure begins with clearly defining the construct of interest and then choosing the methodology best suited to capture it. Simpler, more efficient approaches, such as cross-sectional designs and retrospective reports, remain useful for providing preliminary proof of concept when examining novel environmental features or developmental associations. In Table 2, we provide guidance on how to interpret measures of early experience, including what different measures capture and miss, to assist researchers in selecting appropriate approaches for their specific research questions.

**Table 2:** What different measures capture and miss about early experience.

	**Type of measure**	**What it captures**	**What it misses**
**1**	Cumulative risk scores	• Total dose and co-occurrence of experiences (i.e., their additive effect)	• Nonlinear effects of experiences • Distinct effects of different types of adversity and specific pathways for effects • Variation due to timing and intensity of experience

**2**	Extreme groups	• Presence/absence of exposure to specific experiences	• Variation due to individual differences in nature of focal experience • Consideration of other experiences correlated with the focal experience

**3**	Retrospective reports	• Subjective appraisal and meaning-making of experiences (e.g., which events are “adverse”) • Efficient assessment of lifetime exposure for older children/adults	• Objective exposure • Precise timing and nature of exposure • Early exposure outside of memory

**4**	Prospective reports	• More precise timing and nature of exposure due to reduced recall bias	• Purely objective exposure unless captured in real-time • First-person experiences of infants and young children who do not have capacity to report

**5**	Parent reports of parenting behaviors	• Parental perceptions of their behavior • Insights into dynamics children may not recognize (e.g., monitoring, discipline) • Efficient assessment of multiple perceived behaviors	• Objective frequency and quality of behavior • Behaviors outside parent awareness or that are socially undesirable • Experiences outside of parental awareness

**6**	Cross-informant measures	• Multiple perspectives • Experiences across different contexts (e.g., home, school) • Discrepancies in reporting	• Uncertainty about which perspective is most accurate • Clarity about whether discrepancies reflect true differences in exposure or measurement error

**7**	Administrative data	• Objective documentation of certain exposures (e.g., child protective services records, medical records)	• Events that are not officially documented/reported • Less extreme experiences that don't come to official attention

**8**	Biomarkers	• Objective physiological responses to experiences • Cumulative effects of environment over time (e.g., hair cortisol)	• Experiences themselves • Isolated environmental signal due to multi-determined nature of biomarkers

**9**	Laboratory-based observations	• Standardized, objective assessment of behaviors with a brief observation window • Behaviors in response to controlled conditions	• Typical behaviors in everyday life • Naturalistic variation across contexts and time

**10**	Naturalistic observations	• Objective behaviors in real-world context • Dynamics in daily environment	• Behavior when observer is not present • Behaviors across long time scales (given resource intensiveness of constant observation)

**11**	Passive monitoring	• Objective, real-time measurement • Measures across naturalistic contexts • Temporal dynamics of objective experience • Deep measurement	• Contextual features that moderate effects (e.g., quality vs. quantity of environmental input) • Subjective appraisal and meaning-making of experiences

**12**	Ecological momentary assessment	• Real-time perceptions of experience • Temporal dynamics of subjective experience • Contextual information for passive monitoring measures	• Experiences outside individual awareness or that are socially undesirable

While the choice of measure depends on the research question and application, we propose three minimal criteria for improving insights gained from human research on early experience. First, when selecting measures and interpreting findings, researchers should clearly distinguish between measures of exposure to experiences and measures of psychological or biological responses to those experiences. This distinction is fundamental to establishing construct validity. Second, researchers aiming to identify mechanisms underlying the developmental impact of early experiences should apply dimensional frameworks and use multiple measures to capture distinct aspects of experience (e.g., threat, deprivation, unpredictability, emotional input, cognitive input). While cumulative risk scores remain useful for identifying high-risk subgroups, they obscure the specific pathways through which different experiences confer risk. Third, researchers should measure early experiences continuously or, at a minimum, consider where their focal experience lies along a continuum from enrichment to adversity. Continuous measurement allows for identifying thresholds at which experiences begin to confer risk—or advantages—thereby improving the precision of intervention targets.

## 4 Implications for developmental neuroscience

The measurement challenges discussed here may contribute to inconsistent findings regarding the neural correlates of early adversity, where cumulative risk scores obscure the distinct neural pathways through which different types of experiences (threat vs. deprivation) affect brain development. Addressing these challenges through refined measurement of early experience has profound implications for developmental neuroscience beyond simply identifying individual differences in outcomes. For instance, high-resolution measurement of language input using passive monitoring can reveal critical periods when the brain is most sensitive to linguistic input, informing theories about when neural circuits become specialized for language processing. Similarly, precise measurement of caregiver proximity and responsiveness can clarify how attachment relationships are represented neurally and influence stress-response systems and emotion regulation circuits. Improved measurement also strengthens cross-species translation by enabling researchers to link specific manipulations in animal models (e.g., maternal separation paradigms) to naturalistic variation in human caregiving experiences. By capturing the timing, nature, and intensity of experiences—including through ecologically-valid measures, we can test specific hypotheses about neuroplasticity and directly map environmental inputs onto neural circuit development during critical developmental windows. This precision can also inform the optimal timing of interventions, moving beyond broad recommendations to identify specific developmental windows when particular types of environmental input are most impactful.

## 5 Conclusion

Accurately measuring early experiences is crucial for understanding how the environment shapes brain development but presents significant challenges. Traditional approaches have yielded important insights, but limitations in precision, comprehensiveness, and ecological validity have constrained our ability to capture the complex, multidimensional nature of early experiences and their specific effects on neurodevelopment.

Emerging frameworks that distinguish between different dimensions of adversity, consider timing and sensitive periods, and integrate across levels of analysis offer promising paths forward. Technological advances now make it possible to measure early experiences with greater precision, comprehensiveness, and ecological validity. Addressing genetic confounding through genetically informed designs will be essential for establishing causal links between the environment and development and identifying truly modifiable aspects of children's environments. Future work should also disentangle prenatal from postnatal exposures, as these may have distinct implications for the timing and type of interventions.

By advancing precise, high-dimensional approaches to measuring early experiences, researchers can improve understanding of fundamental neurodevelopmental processes while also addressing questions of practical significance in education, mental health, and social policy. This dual focus on basic and applied science—enabled by methodological advances in passive monitoring, dimensional frameworks, and genetically-informed designs—makes refining the measurement of early experiences a priority for developmental neuroscience.

## CRediT authorship contribution statement

**Kathryn L Humphreys:** Writing – review & editing, Writing – original draft, Conceptualization. **Lucy S. King:** Writing – review & editing, Writing – original draft, Conceptualization.

## Funding

This work was supported by the 10.13039/100000001National Science Foundation [Grant no. 2042285] and the 10.13039/100000025National Institute of Mental Health [Grant no. R01MH129634]; both awarded to Kathryn L. Humphreys.

## Declaration of Competing Interest

The authors declare that they have no known competing financial interests or personal relationships that could have appeared to influence the work reported in this paper.

## Data Availability

No data was used for the research described in the article.
